# Clinical and CT characteristics of Xpert MTB/RIF-negative pulmonary tuberculosis

**DOI:** 10.1371/journal.pone.0250616

**Published:** 2021-05-03

**Authors:** Han Na Lee, Jung Im Kim, Yee Hyung Kim

**Affiliations:** 1 Department of Radiology, Kyung Hee University Hospital at Gangdong, Kyung Hee University School of Medicine, Seoul, Republic of Korea; 2 Department of Respiratory Medicine, Kyung Hee University Hospital at Gangdong, Kyung Hee University School of Medicine, Seoul, Republic of Korea; School of Medicine, Tehran University of Medical Sciences, ISLAMIC REPUBLIC OF IRAN

## Abstract

**Purpose:**

To determine the diagnostic accuracy of the Xpert MTB/RIF assay in patients with smear-negative pulmonary tuberculosis (TB) and to assess clinical and CT characteristics of Xpert-negative pulmonary TB.

**Material and methods:**

We retrospectively reviewed the records of 1,400 patients with suspected pulmonary TB for whom the sputum Xpert MTB/RIF assay was performed between September 1, 2014 and February 28, 2020. Clinical and CT characteristics of smear-negative pulmonary TB patients with negative Xpert MTB/RIF results were compared with positive results.

**Results:**

Of 1,400 patients, 365 (26.1%) were diagnosed with pulmonary TB and 190 of 365 patients (52.1%) were negative for sputum acid-fast bacilli. The diagnosis of pulmonary TB was based on a positive culture, positive Xpert MTB/RIF or the clinical diagnoses of patients treated with an anti-TB medication. The sensitivity, specificity, positive predictive and negative predictive values of sputum Xpert MTB/RIF for smear-negative pulmonary TB were 41.1%, 100%, 100%, and 90.1%, respectively. Finally, 172 patients with smear-negative pulmonary TB who underwent chest CT within 2 weeks of diagnosis were included to compare Xpert-positive (n = 66) and Xpert- negative (n = 106) groups. Patients with sputum Xpert-negative TB showed lower positive rates for sputum culture (33.0% vs. 81.8%, p<0.001) and bronchoalveolar lavage culture (53.3% vs. 84.6%, p = 0.042) than in Xpert-positive TB. Time to start TB medication was longer in patients with Xpert-negative TB than in Xpert-positive TB (11.3±16.4 days vs. 5.0±8.7 days, p = 0.001). On chest CT, sputum Xpert-negative TB showed significantly lower frequency of consolidation (21.7% vs. 39.4%, p = 0.012), cavitation (23.6% vs. 37.9%, p = 0.045), more frequent peripheral location (50.9% vs. 21.2 p = 0.001) with lower area of involvement (4.3±4.3 vs. 7.6±6.4, p<0.001). Multivariate analysis revealed peripheral location (odds ratios, 2.565; 95% confidence interval: 1.157–5.687; p = 0.020) and higher total extent of the involved lobe (odds ratios, 0.928; 95% confidence interval: 0.865–0.995; p = 0.037) were significant factors associated with Xpert MTB/RIF-negative TB. Regardless of Xpert positivity, more than 80% of all cases were diagnosed of TB on chest CT by radiologists.

**Conclusion:**

The detection rate of sputum Xpert MTB/RIF assay was relatively low for smear negative pulmonary TB. Chest CT image interpretation may play an important role in early diagnosis and treatment of Xpert MTB/RIF-negative pulmonary TB.

## Introduction

Tuberculosis (TB) remains a critical cause of death among infectious disease worldwide. In 2018, 10 million people fell ill and over 1.5 million people died of TB, which is a preventable and curable disease [1]. Therefore early detection and appropriate treatment are important for effective control of TB transmission.

For TB diagnosis, sputum acid-fast bacilli (AFB) smear is easy to perform, but it shows low sensitivity of 50–70% for culture positive TB [2, 3]. Although culture is considered the reference standard for TB diagnosis, it is time-consuming and takes 6 and 8 weeks on liquid and solid culture media, respectively [4, 5]. Therefore molecular diagnostic methods, such as real-time polymerase chain reaction (PCR) assay and Xpert MTB/RIF (Cepheid; Sunnyvale, USA) assay, have been developed and are widely used for early detection of TB with higher sensitivity.

The Xpert MTB/RIF assay was endorsed by the World Health Organization (WHO) in December 2010 [6]. It can simultaneously identify *Mycobacterium tuberculosis* and rifampin resistance within 2 hours using automated single cartridge-based nucleic acid amplification [7]. According to recent meta-analysis, Xpert MTB/RIF assay has high sensitivity of 99% for smear-positive patients and 57–83% for smear-negative TB [8, 9]. In January 2020, WHO highlighted the importance of Xpert MTB/RIF assay. It has provided high diagnostic accuracy and improved patient-important outcomes when it was used as an initial test for TB and rifampicin-resistant TB diagnosis, replacing smear microscopy [10].

Although Xpert MTB /RIF assay has developed TB diagnosis, it is known to be less sensitive in detecting AFB smear-negative TB, culture-negative TB, and for patients in countries with high TB prevalence [8]. South Korea is a country with an intermediate incidence of TB [11]. Previous studies showed a high diagnostic sensitivity of Xpert MTB/RIF for bronchoscopic specimen with 80–82% in our country [7, 12], but the diagnostic accuracy of Xpert MTB/RIF for sputum specimen, which are usually tested by initial, has not been known.

Pulmonary TB is considered when patients have clinical, bacteriological, and/or, radiographic evidence of current tuberculosis [13]. Abnormal image findings suggestive of pulmonary TB may be important, especially when the results of ABF smear and molecular assay are all negative. We thought that CT image findings related with sputum Xpert MTB/RIF-negative pulmonary TB maybe helpful for TB diagnosis, mentioned above. Previous study compared CT findings of pulmonary TB between sputum PCR positive and negative, but they included relatively small cases, and there is no mention of whether they used one method of molecular assay [14].

Therefore this study aimed to evaluate the diagnostic performance of sputum Xpert MTB/RIF in suspected pulmonary TB patients with smear-negative and to evaluate the clinical and CT characteristics of sputum Xpert MTB/RIF-negative TB compared with Xpert MTB/RIF-positive TB in adult patients.

## Materials and methods

### Patient selection

This study was approved by the Institutional Review Board of Kyung Hee University Hospital at Gangdong (2020-09-024), and the need for informed consent was waived. Patients’ medical records were fully anonymized before the 6-months examination period. We retrospectively reviewed the medical records of all adult patients with clinically suspicious pulmonary TB for whom sputum smears for AFB and sputum Xpert/MTB assay were performed between September 1, 2014 and February 28, 2020.

Diagnosis of pulmonary TB was defined by the culture of M. *tuberculosis* from sputum or other specimens, such as bronchoscopic specimen or lung tissue obtained by percutaneous transthoracic needle biopsy. Culture-negative pulmonary TB was defined as patients with suspected radiologic and respiratory symptoms and negative culture for M. *tuberculosis* for three initial sputum or other specimens [15] and who met one of the following inclusion criteria: (1) positive TB-PCR on sputum and/or bronchoscopic specimen and/or lung tissue, and (2) radiologic improvement on anti-tuberculosis medication. Patients with loss to follow-up during TB medication for culture-negative TB and who took TB medication before examination of Xpert MTB/RIF assay were excluded from the analysis.

To compare clinical and CT characteristics of Xpert MTB/RIF negative-pulmonary TB with Xpert MTB/RIF positive-pulmonary TB in adult patients, we only selected patients with negative sputum AFB and those who performed chest CT within 14 days of TB diagnosis among the above TB patients. Clinical information, such as patients’ demographics, body mass index, smoking status, comorbidities, history of TB treatment, and quantiferon results, were evaluated. Time periods to take initial TB medication were also recorded. If they were concurrently tested for real-time TB-polymerase chain reaction, results were reviewed. For patients who performed bronchoscopy for TB diagnosis, its related information such as the Xpert MTB/RIF and culture for bronchoalveolar lavage (BAL) specimen was also evaluated.

### Xpert MTB/RIF assay, acid-fast bacilli smear, and mycobacterial culture

The Xpert MTB/RIF (Cepheid, Sunnyvale, CA, USA) can detect TB and rifampin resistance simultaneously within 2 hours using RT-PCR for the TB-specific *rpoB* gene. The Xpert assay was performed according to the manufacturer’s instructions. The Xpert MTB/RIF reagent was added to the untreated sputum with a 2:1(v/v) ratio. The sample mixture with 2mL was incubated for 15 minutes at room temperature with vigorous agitation and then transferred into an Xpert cartridge before insertion into the GeneXpert instrument [16].

AFB smears were stained with the auramine-rhodamine fluorochrome stain and microscopically examined under 200x magnification and confirmed by Ziehl-Neelsen staining.

For culture of *M*. *tuberculosis*, the sediment was incubated on 3% Ogawa solid medium (Asan pharmaceutical, Seoul, Korea) for 8 weeks and BACTEC^™^ MGIT^™^ system, a liquid culture (BD, Sparks, MD, USA) for 6 weeks. Once cultured, isolation of MTB was tested by Cobas Taqman 48 MTB test (Roche Diagnostics, Mannheim, Germany) and/or TB Ag MPT64 Rapid (Standard Diagnostics, Inc., Kyonggi-do, Korea). All procedures were performed according to the manufacturer’s instructions.

### Chest CT acquisition and analysis of CT findings

Chest CT with or without an intravenous injection of contrast medium were obtained with two multidetector CT. Parameters for a 64 MDCT (Brilliance 64 CT scanner; Philips Medical Systems, Cleveland, OH) were 120 kVp, 30–200 mAs with automatic tube current modulation, collimation of 0.625 mm, pitch of 1.105, gantry rotation time of 0.5 second, and matrix size of 512 x 512. Parameters for a 256 MDCT (Revolution CT, GE Healthcare, Milwaukee, WI, USA) were 120 kVp, 30–300 mAs, noise index of 20 with automatic tube current modulation, collimation of 0.625 mm, gantry rotation time of 0.5 second, and matrix size of 512 x 512.

Chest CT imaging features were analyzed by two thoracic radiologists with 15 and 10 years of experience and final decision was made in consensus. They were aware of the diagnosis of TB but were blinded to results of Xpert MTB/RIF and patients’ demographic and clinical features.

Parenchymal abnormalities were assessed for the presence of centrilobular nodules, perilymphatic nodules, random distributed nodules, tree-in-bud pattern, consolidation, cavity, macronodules, and bronchiectasis. The presence of lymphadenopathy, pleural effusion, and pleural thickening were also reviewed. Parenchymal abnormal findings were defined based on the nomenclature provided by Fleishner Society [17].

Lung parenchyma was divided into six zones (upper, mid, lower on each side) based on the levels of the tracheal carina and the right inferior pulmonary vein as anatomical landmark for the division of upper and mid lung zone, and the division of mid and lower lung zone respectively). The extent of lung abnormalities was scored using a five point scale for each six zones: 0, no involvement; 1, 1–25% involvement; 2, 26–50% involvement; 3, 51–75% involvement; and 4, 76–100% involvement. The total extent was then defined by the sum of five point scale of six zones. In addition, the number of involved lobe and only one segment involvement were also recorded.

As for the distribution of parenchymal abnormalities, axial distribution (central, peripheral, mixed) and longitudinal distribution (upper, lower, mixed) were evaluated. The inner two-third of the lung was defined as central and the outer third of the lung was defined as peripheral. For longitudinal distribution, the lesions above the hilum were considered as upper and those below the hilum were regarded as lower. The lesions that involved the lung parenchyma without dominant distribution were considered to have mixed distributions. Finally, CT interpretation by radiologists were divided into three categories; TB diagnosis as first impression, TB diagnosis included for differential diagnosis, and TB not included in report.

### Statistical analysis

Continuous variables were expressed as the mean and standard deviation and categorical variables were represented as counts and percentages. Kolmogorov–Smirnov test was used to assess the normality of distribution.

All comparisons between Xpert MTB/RIF negative and positive TB were analyzed using an independent samples t tests or the Mann–Whitney U test for continuous variables and using the chi-square or Fisher’s exact statistics for categorical variables. To determine clinical and radiologic factors related with false-negative Xpert MTB/RIF results for sputum specimen and false-negative Xpert MTB/RIF results for BAL specimen, multivariate logistic analysis was performed. Variables with p<0.05 at univariate analysis were used as input variables for multivariate analysis.

The sensitivity, specificity, positive predictive value (PPV), and negative predictive value (NPV) were calculated using the 95% confidence intervals (CI) to assess the diagnostic accuracy of Xpert MTB/RIF assay for sputum specimen.

Statistical analysis was performed using SPSS (v. 18.0, IBM Inc, Chicago, Ill, USA) and SAS (v. 9.4, SAS Institute, Cary, NC, USA).

## Results

### Patient selection

**Fig 1:**
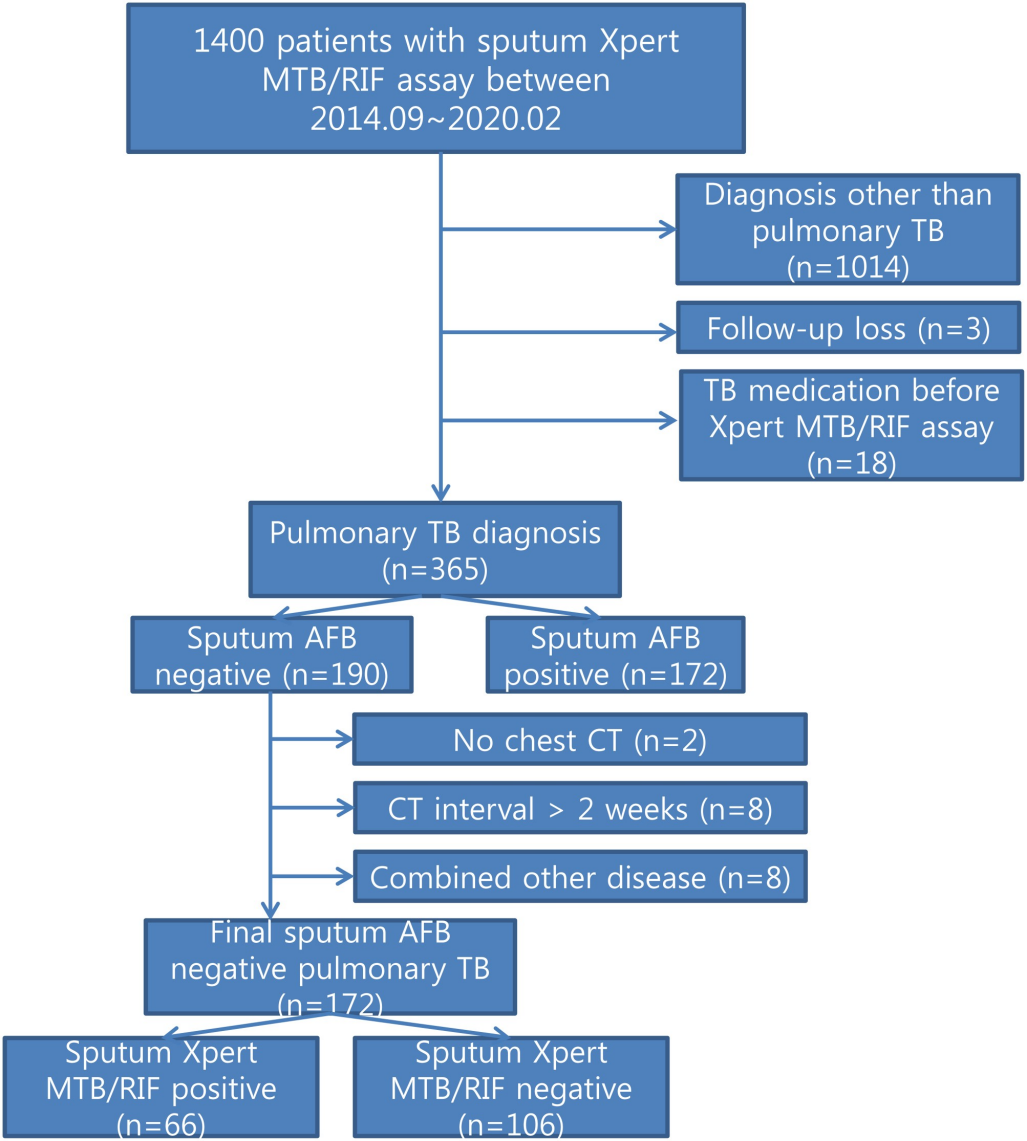
Patient selection for comparison of smear-negative pulmonary TB between Xpert MTB/RIF positive and negative.

A total of 1400 patients with clinically suspicious pulmonary TB performed sputum smears for AFB and sputum Xpert MTB/RIF assay simultaneously. Among them, 386 patients were diagnosed with pulmonary TB based on the presence of positive cultures or Xpert MTB/RIF or a clinical diagnosis based on the administration of anti-TB medication. There were 1,014 patients diagnosed with diseases other than pulmonary TB. These included 58 extrapulmonary TB cases, and 956 patients who did not have any form of TB, but who were diagnosed with other diseases such as pneumonia, bronchiectasis/bronchiolitis, empyema and nontuberculous mycobacteria. Twenty-one patients were excluded for further analysis, because of loss to follow up (n = 3) and TB medication start before Xpert MTB/RIF assay (n = 18). Among 365 patients, 190 patients were negative for sputum AFB. For comparison of clinical and CT findings between sputum Xpert MTB/RIF positive and negative, we excluded 18 patients who did not performed chest CT (n = 2) or performed chest CT with more than 2 weeks of interval within diagnosis of TB (n = 8) and patients who have concurrent diffuse parenchymal disease on chest CT (n = 8). Finally, 172 patients including 66 patients with Xpert MTB/RIF positive and 106 patients with Xpert MTB/RIF negative were included (Fig 1).

### Diagnostic performance of sputum Xpert MTB/RIF

Diagnostic performance of sputum Xpert MTB/RIF for smear-negative pulmonary TB is presented in Table 1. The sensitivity, specificity, positive predictive value negative predictive value, and accuracy of sputum Xpert MTB/RIF for diagnosis of pulmonary TB was 41.05% (95% CI, 33.98–48.40), 100.00% (95% CI, 99.64–100.00) 100.00%, 90.05 (95% CI, 88.91–92.28), and 90.70%, respectively. When analyzed the diagnostic performance based on only culture positive pulmonary TB, the sensitivity, specificity, positive predictive value, negative predictive value, and accuracy was 50.00% (95% CI, 41.04–58.96), 98.70% (95% CI, 97.83–99.29), 82.05% (95% CI, 72.54–88.78), 94.32% (95% CI, 93.31–95.18) and 93.52%, respectively.

**Table 1:** Diagnostic performance of sputum Xpert MTB/RIF for smear-negative pulmonary TB.

Pulmonary TB for smear-negative	Xpert MTB/RIF		Xpert MTB/RIF^a^	
	%(n/N)	%(95% CI)	n/N	%(95% CI)
**Sensitivity**	41.05 (78/190)	33.98–48.40	50.00 (64/128)	41.04–58.96
**Specificity**	100.00 (1014/1014)	99.64–100.00	98.70 (1062/1076)	97.83–99.29
**Positive predictive value**	100.00 (78/78)	-	82.05 (64/78)	72.54–88.78
**Negative predictive value**	90.05 (1014/1126)	88.94–91.07	94.32 (1062/1126)	93.31–95.18
**Accuracy**	90.70	88.91–92.28	93.52	91.98–94.85

Note-TB = tuberculosis, CI = confidence interval.

^a^. Analysis was performed for pulmonary TB cases that was diagnosed only based on culture positivity.

### Clinical characteristics of patients with sputum Xpert MTB/RIF negative and positive

The demographic and clinical characteristics of the included pulmonary TB patients who were negative for sputum AFB and performed sputum Xpert MTB/RIF assay are shown in Table 2. Demographic characteristics were not significantly different between Xpert MTB/RIF negative and Xpert MTB/RIF positive pulmonary TB.

**Table 2:** Clinical characteristics of patients with Xpert MTB/RIF positive and negative.

	Total patients (n = 172)	Xpert-negative (n = 106)	Xpert-positive (n = 66)	P-value
Age	52.0±20.4	50.3±19.1	54.6±22.4	0.180^a^
Sex				0.268
Male	118(68.6)	76(71.7)	42(63.6)	
Female	54(31.4)	30(28.3)	24(36.4)	
Body mass index (kg/m2)	21.2±3.2	21.2±3.1	21.2±3.3	0.939^a^
Smoking status				0.621
Never smoker	94(54.7)	57(53.8)	37(56.1)	
Ex-smoker	35(20.3)	20(18.9)	15(22.7)	
Current smoker	43(25.0)	29(27.4)	14(21.2)	
Comorbidities				0.489
Yes	47(27.3)	27(25.5)	20(30.3)	
Previous TB treatment	19(11.0)	10(9.4)	9 (13.6)	0.393
Quantiferon positive	72/80(90.9)	49/53(92.5)	23/27(85.2)	0.258^b^
TB diagnosis				<0.001^b^
Culture positive	115(66.9)	59(55.7)	56(84.8)	
Pathologic diagnosis	11(6.4)	11(10.4)	0	
Clinical diagnosis	35(20.3))	35(33.0)	0	
Bronchial Xpert only	2(1.2)	1(0.9)	1(1.5)	
Sputum Xpert only	9(5.2)	0	9(13.6)	
Culture-positive sputum	89(51.7)	35(33.0)	54(81.8)	<0.001
BAL	58(33.7)	45(41.7)	13(19.7)	0.002
BAL Xpert positive	36/58(62.1)	25/45(55.6)	11/13(84.6)	0.053^b^
BAL culture positive	35/58(60.3)	24/45(53.3)	11/13(84.6)	0.042
TB PCR detection rate	10/51(19.6)	3/29(10.3)	7/22(31.8)	0.060^b^
TB medication start periods	9.0±14.2	11.3±16.4	5.0±8.7	0.001^a^

Note-BAL = bronchoalveolar lavage.

All data are presented in number (%) or mean±standard deviation

^a^. Independent samples t-test,

^b^. Fisher’s exact test, except where indicated Chi-square test

Among 172 patients with pulmonary TB, 115 patients (66.9%) had culture-confirmed pulmonary TB with sputum, bronchial washing and/or pathologic specimens. Eleven patients (6.4%) were diagnosed with pathological findings, nine patients (5.2%) through sputum Xpert MTB/RIF positive only, and two patients (1.2%) through Xpert MTR/RIF positive for BAL specimen only. Thirty-five patients (20.3%) were diagnosed clinically with the evidence of improvement after treatment with anti-tuberculosis medication.

Culture positive rate for sputum was significantly lower in patients with Xpert MTB/RIF negative TB compared to positive TB (33.0% vs. 81.8%, p<0.001). Compared to the patients with Xpert MTB/RIF positive TB, Xpert MTB/RIF negative TB were also less likely to show positive results for BAL culture (53.3 vs. 84.6%, p = 0.042) and for BAL Xpert MTB/RIF (55.6% vs. 84.6.%, p = 0.053), but the latter was not statistically significant.

TB medication start periods in patients with Xpert MTB/RIF negative were longer than that in patients with Xpert MTB/RIF positive (11.3 days vs. 5.0 days, p = 0.001).

### Chest CT findings comparison between sputum Xpert MTB/RIF negative vs. positive

**Fig 2:**
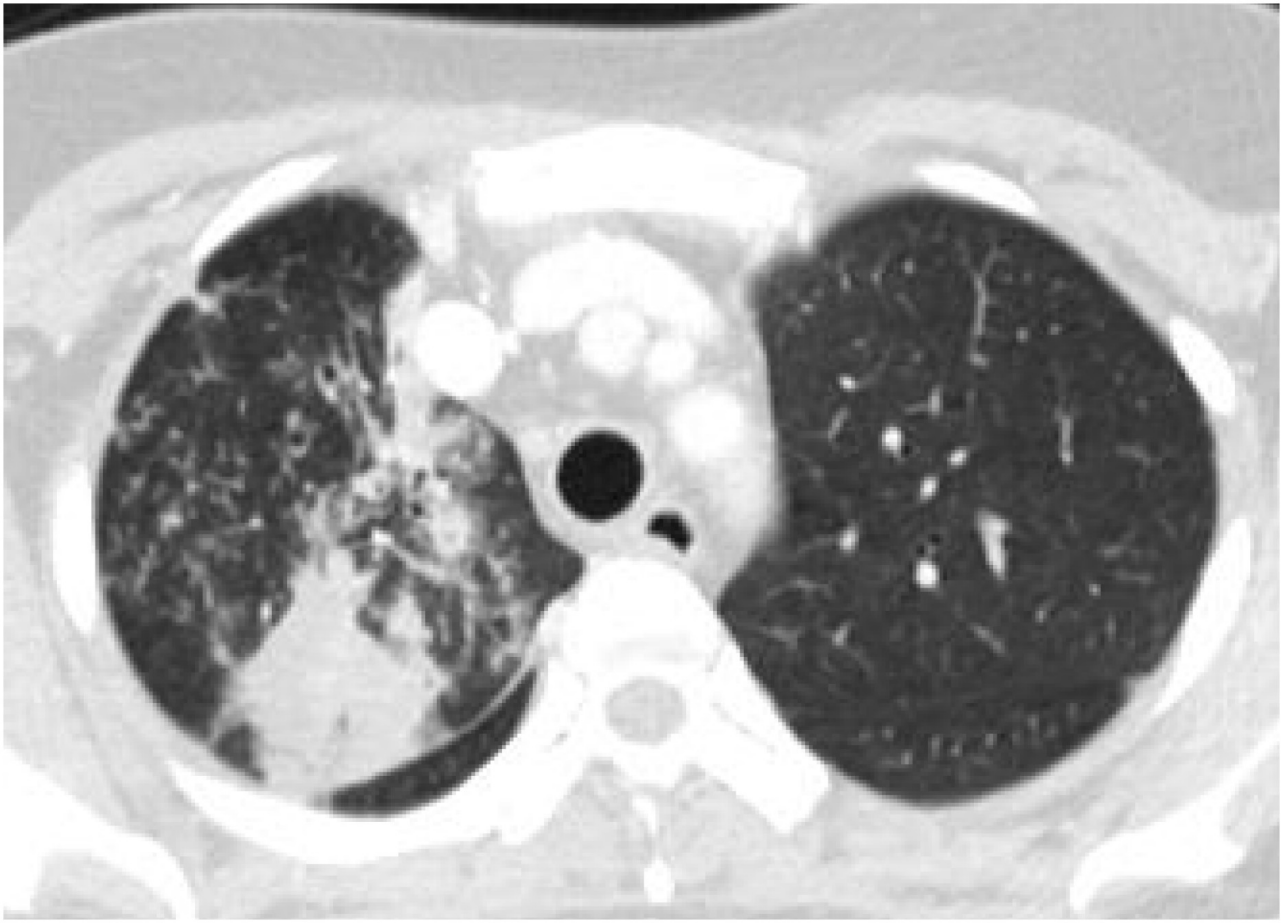
A 37-year-old woman with pulmonary TB with positive sputum MTB/RIF. Sputum acid-fast bacilli was negative, but sputum Xpert MTB/RIF was positive. Chest CT shows consolidation with multiple centrilobular nodules in the right upper lobe. Rapid TB medication was initiated and sputum culture also showed positive later.

**Fig 3:**
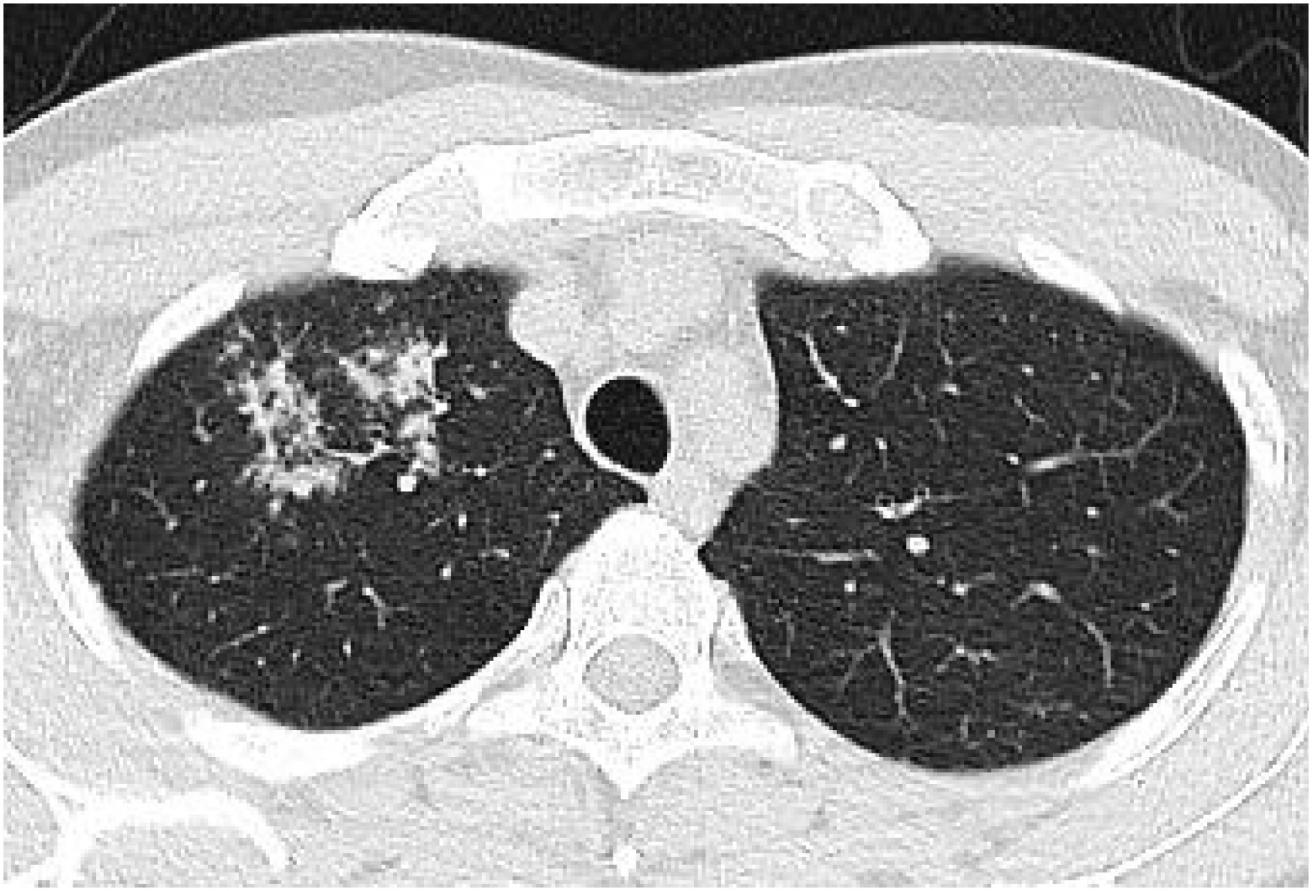
A 30-year-old man with pulmonary TB with negative sputum MTB/RIF. Chest CT shows centrilobular nodules confined to the apical segment of the right upper lobe with peripheral location. Sputum Xpert MTB/RIF was negative, but he was confirmed as active pulmonary TB after positive Xpert MTB/RIF assay on bronchoalveolar lavage.

Xpert MTR/RIF negative pulmonary TB were less likely to show consolidation (21.7% vs. 39.4%, p = 0.012) and cavity (23.6% vs. 37.9%, p = 0.045) with more peripheral axial distribution (50.9% vs. 21.2%, p<0.001), compared to Xpert positive TB. Other chest CT findings, such as centrilobular nodules, perilymphatic nodules, random distributed nodules, tree-in-bud, and macronodules, did not show statistical differences between them (all, p>0.05) (Table 3). Regarding extent, the number of involved lobe (1.9±1.5 vs. 2.94±1.9, p<0.001) and total extent (4.3±4.3% vs. 7.6±6.4%, p<0.001) were significantly lower in Xpert MTB/RIF negative TB compared to Xpert MTB/RIF positive TB. Xpert MTB/RIF negative TB showed higher incidence of only one segment involvement compared to Xpert MTB/RIF positive TB (23.6% vs. 10.6%, p = 0.033) (Figs 2 and 3). Regardless of Xpert positivity, more than 80% of all cases were diagnosed of TB on chest CT by radiologists. When performing multivariate logistic regression analysis, peripheral location (odds ratios, 2.565; 95% confidence interval: 1.157–5.687; p = 0.020), and higher total extent of the involved lobe (odds ratios, 0.928; 95% confidence interval: 0.865–0.995; p = 0.037) were significant independent factors for negative sputum Xpert MTR/RIF results in patients with pulmonary TB. The Hosmer-Lemeshow goodness-of-fit test was 0.923.

**Table 3:** Comparison of chest CT findings between Xpert MTB/RIF negative and positive.

	Xpert-negative (n = 106)	Xpert-positive (n = 66)	P-value
Centrilobular nodules	91(85.8)	57(86.4)	0.925
Perilymphatic nodules	8(7.5)	3(4.5)	0.534^a^
Random nodules	1(0.9)	2(3.0)	0.559^a^
Tree-in-bud	43(40.6)	29(43.9)	0.663
Consolidation	23(21.7)	26(39.4)	0.012
Cavity	25(23.6)	25(37.9)	0.045
Macronodules	49(46.2)	24(36.4)	0.203
Bronchiectasis	11(10.4)	10(15.2)	0.352
Lymphadenopathy	15(14.2)	11(16.7)	0.654
Pleural effusion	13(12.3)	11(16.7)	0.418
Pleural thickening	9(8.5)	6(9.1)	0.892
Axial distribution			0.001^a^
Central	0	0	
Peripheral	54(50.9)	14(21.2)	
Mixed	52(49.1)	52(78.8)	
Longitudinal distribution			0.087
Upper	73(68.9)_	35(53.0)	
Lower	10(9.4)	7(10.6)	
Mixed	23(21.7)	24(36.4)	
Number of involved lobe	1.9±1.5	2.9±1.9	<0.001^b^
One segment involvement	25(23.6)	7(10.6)	0.033
Total extent	4.3±4.3	7.6±6.4	<0.001^b^
Radiologic diagnosis of TB			0.690^a^
first impression	91(85.8)	55(83.3)	
differential diagnosis	10(9.4)	9(13.6)	
no TB diagnosis	5(4.7)	2(3.0)	

All data are presented in number (%) or mean±standard deviation

^a^. Fisher’s exact test, except,

^b^. Independent samples t-test, except where indicated Chi-square test

### Chest CT findings comparison between bronchoalveolar lavage Xpert MTB/RIF negative vs. positive

Chest CT findings were compared between patients with Xpert MTB/RIF negative (n = 22) and Xpert MTR/RIF positive (n = 35) for BAL specimen. Patients with Xpert MTB/RIF negative for BAL specimen were less likely to show cavity than Xpert MTB/RIF positive (13.6% vs. 42.9%, p = 0.021) and other parenchymal abnormal findings did not show statistical differences between the two groups (all, p>0.05). Axial and longitudinal distribution were not significantly different (p = 0.239 and p = 0.279, respectively). The number of involved lobe (1.6±1.2 vs. 2.8±1.9, p = 0.004) and the total extent (3.6±3.9% vs. 7.3±6.8%, p = 0.013) were significantly lower in Xpert MTB/RIF negative TB for BAL specimen compared to positive. When multivariate logistic regression analysis was conducted for 57 patients, higher total extent of involved lobe (odds ratios, 0.889; 95% confidence interval: 0.805–0.981; p = 0.019) was a negatively associated factor for BAL negative Xpert MTB/RIF. The Hosmer-Lemeshow goodness-of-fit test was 0.787.

## Discussion

Xpert MTB/RIF test has developed rapid diagnosis of pulmonary TB and MDR-TB cases [18]. We evaluated the diagnostic yield of sputum Xpert MTB/RIF in suspected smear-negative TB patients, because the results of sputum Xpert MTB/RIF for smear-negative patients is more relevant in clinical practice. To the best of our knowledge, this is the first study to compare CT findings of pulmonary TB cases according to sputum Xpert MTB/RIF positivity. We also suggested CT imaging findings associated with false-negative TB, which may help to decide whether further work-up is needed or whether it should be treated with anti-TB medication for patients with suspected TB, but who have negative results on the sputum AFB and Xpert MTB/RIF tests.

Our result revealed that sensitivity of the Xpert MTR/RIF was 46.0% for TB cases including clinical diagnosis of TB and 50.0% for only culture-proven TB cases. Previous studies showed variable results of sensitivity of Xpert MTB/RIF ranging from 47% to 87% for smear-negative patients [8, 19–23]. In a previous meta-analysis, the pooled sensitivity for smear-negative and culture-positive TB was 67%-lower than the 98% pooled sensitivity for smear-positive TB [18]. Another meta-analysis revealed sensitivity of 70% for smear-negative and culture-proven TB and 52% for smear-negative and clinical diagnosis of TB [8]. Sensitivity depends on the definition of TB diagnosis, smear status, varing populations, and test strategies [8, 18]. Comparatively, our data revealed a relatively lower sensitivity. There are several possible explanations for this finding. Our population had an intermediate TB burden and low HIV-prevalence [8]. Second, it is possible that some sputum specimens were not well expectorated by patients with TB. Lastly, more early-stages TB lesions—undetectable by sputum Xpert MTB/RIF—may have been included in our study, since CT scans are widely used and easily performed with low cost by health insurance in our country, which may have increased the detection rate of early-stage pulmonary TB [24].

In this study, CT findings of consolidation and cavitation were more frequently observed in patients with sputum Xpert MTB/RIF positive than in patients with negative and Ko et al. [14] reported the same results. In addition, it is known that the presence of consolidation and cavity were also related with the number of AFB on sputum smear or smear-positive pulmonary TB [25, 26]. These results attributed to large amounts of bacilli excreted in TB patients showing consolidation and cavitation. However, these CT findings were not a significant factor in multivariate analysis, which could be because we compared image findings between Xpert MTB/RIF positive and negative for only patients with smear-negative. Further study is needed to clarify these findings related to positivity of Xpert MTB/RIF.

Peripheral location and lower total extent of involvement remained a significant factor associated with negative Xpert MTR/RIF TB. Peripheral location, which means that longer distance between the involved area and central airway, maybe related with sputum-negative Xpert MTB/RIF. We found no study comparing the extent of CT findings between sputum Xpert MTB/RIF positive and negative. In this study, the average total extent score in Xpert MTR/RIF negative group was 3.3 point (an extent equivalent to 11.0%) lower than that in the Xpert positive group. These findings of TB suggests a lower mycobacterial burden and is usually found in the early stage of active pulmonary TB which shows small centrilobular nodules with peripheral location and a lower extent of involvement [27, 28].

Bronchoscopy has been a useful tool for the diagnosis of pulmonary TB in patients who cannot produce sputum or those who are sputum smear and Xpert MTR/RIF negative [29]. The sensitivity of the Xpert MTR/RIF from BAL specimen ranged from 80%-93% in previous studies [7, 30, 31] and lower sensitivity of 60–73% was reported in patients with smear-negative TB [32, 33]. In this study, when BAL was performed for patients with sputum Xpert MTB/RIF negative, about 50% of them could obtain positive Xpert MTR/RIF results for BAL specimen. According to our study, lower extent on chest CT increased the possibility of negative results when BAL was additionally performed.

This study showed more than 80% correct interpretation of pulmonary TB on chest CT by radiologists, regardless of sputum Xpert MTB/RIF results. Previous study also revealed that CT interpretation, suggestive of pulmonary TB was 81.1% when sputum smear was negative [34]. Therefore this study shows the importance of chest CT scans for TB diagnosis, when sputum Xpert MTB/RIF is negative.

As expected, there was a significant difference in the treatment start periods according to Xpert MTR/RIF positivity. In the case of Xpert MTR/RIF negative, the start of treatment was delayed by an average of 6 days compared to the Xpert MTR/RIF positive, and there was a case of delay of up to 27 days. The decision to initiate TB treatment empirically prior to microbiologic confirmation is based on clinical, radiographic, laboratory, patient, and public health factors. Among them molecular test results and radiologic image findings are included factors to be considered [26]. Therefore our study results of clinical and CT features related with negative Xpert MTR/RIF TB could be helpful for decision of empirical treatment.

This study has some limitations. First retrospective study included only patients who performed sputum AFB and sputum Xpert MTR/RIF at the single medical center. Second, because sputum specimens were collected in this study, it can produce the heterogeneity of specimens; however, the sputum Xpert MTR/RIF is a test first performed for patients with suspected tuberculosis and the result determines which further diagnostic procedure should be performed. Third, lack of absolute certainty for the clinical diagnosis of TB in which patients improved on anti-tuberculosis treatment.

In conclusion, the detection rate of sputum Xpert MTR/RIF assay was relatively low with 41.0% for smear negative pulmonary TB. Xpert negative pulmonary TB showed more peripheral location and lower extent of involvement compared to Xpert positive pulmonary TB. Chest CT image interpretation may have an important role for early diagnosis and treatment of Xpert MTR/RIF-negative pulmonary TB.

## Supporting information

S1 Data (XLSX)
